# Supplementary material for: Using Ex Situ Seedling Baiting to Capture Seedling-Associated Mycorrhizal Fungi in Medicinal Orchid Dendrobium officinale
Source: J Fungi (Basel). 2022 Sep 29;8(10):1036. doi: 10.3390/jof8101036 (PMC9605586; doi:10.3390/jof8101036)
Supplement: Supplementary file 1 [file jof-08-01036-s001.zip › jof-1929749-supplementary.pdf]

Table S1.

The list of orchid mycorrhizal fungi isolated from *Dendrobium* species with the sampling sites and original resources.

Fungi species (GenBank accession No.)	Sampling sites	*Dendrobium* species	Sources	References
Tulasnella sp. TPYD-1 (MN545675)<br>*Tulasnella* sp. TPYD-2 (MN545849)<br>*Tulasnella* sp. TPYD-3 (MN545858)<br>*Tulasnella* sp. TPYD-4 (MN544859)<br>*Tulasnella* sp. TPYD-5 (MN544860)	Guizhou	*D. officinale*	Seedling roots	[19]
Tulasenlla sp. FDaI1 (KC796459)<br>*Tulasenlla* sp. FDaI4 (KC796460)<br>*Tulasenlla* sp. FDaI5 (KC796462)<br>*Tulasenlla* sp. FDaI6 (KC796461)<br>*Tulasenlla* sp. FDaI7 (KC796463)<br>*Tulasenlla* sp. FDaI8 (KC796464)<br>*Tulasenlla* sp. FDaI9 (KC796465)	Yunnan	*D. aphyllum*	Protocorms	[24]
Epulorhiza sp. FDd1	Yunnan	*D. devonianum*	Protocorms	[25]

Tulasnella sp. DMG1 (MN173009)<br>*Tulasnella* sp. DMG2 (MN173010)<br>*Tulasnella* sp. DMG3 (MN173012)<br>*Tulasnella* sp. DMG4 (MN173013)<br>*Tulasnella* sp. DMG5 (MN173014)<br>*Tulasnella* sp. DMG6 (MN173015)	Yunnan	*D. moniliforme*	Protocorms	[27]
Tulasnella sp. DesI (MK555326)<br>*Tulasnella* sp. DerIV (MK555331)<br>*Tulasnella* sp. DerV (MK555332)	Yunnan	*D. exile*	Roots	[28]
Tulasnella calospora TG1(MN607233)<br>*Tulasnella calospora* TG3 (MN607235)<br>*Tulasnella calospora* TY3 (MN607230)<br>*Tulasnella calospora* JM (MN607232)<br>Tulasnellaceae TY2 (MN607229)<br>*Tulasnella* sp. TG2 (MN607234)<br>*Tulasnella* sp. TY4 (MN607231)<br>Sebacinaceae TY1 (MN607228)<br>Sebacinales LQ (MN173026)	Chongqing	*D. officinale*	Protocorms or root	[29]
Tulasnella sp. CY (KM226996)	Yunnan	*D. devonianum*	Protocorms	[30]

Epulorhiza sp. 3564 (HQ853685)<br>Ceratobasidiaceae 5168 (JF799768)<br>Ceratobasidiaceae 5170 (JF799769)<br>Sebacinaceae 5390 (JF799764)<br>Sebacinaceae 3597 (JF799766)<br>Sebacinaceae 3601 (JF799767)<br>Sebacinaceae 5173 (JF799765)<br>Sebacinales 4035 (JF906112)	Yunnan or Guizhou	*D. nobile* or *D.chrysanthum*	Protocorms or Root	[60]
Mycena dendrobii 05	Yunnan	*D. officinale*	Root	[61]
Epulorhiza sp. DC-3<br>*Epulorhiza* sp. DC-4<br>*Epulorhiza* sp. DC-8<br>*Ceratorhiza* sp. DC-9<br>*Rhizoctomia* sp. DC-12<br>*Mycena dendrobii*. DC-13	Sichuan	*D. officinale*	Root	[62]
Serendipitaceae JC-01 (MH500251)<br>Serendipitaceae JC-02 (MH500252)<br>Serendipitaceae JC-04 (MH500254)<br>Serendipitaceae JC-05 (MH500255)<br>Tulasnellaceae JC-03 (MH500253)	Yunnan	*D. nobile*	Protocorms	[63]

Tulasnella sp.GC-11 (MK182987)<br>*Tulasnella* sp.GC-14 (MK182989)<br>*Tulasnella* sp.GC-15 (MK182987)	Yunnan	*D. chrysotoxum*	Protocorms	[64]
Tulasnellaceae SSCDO-1 (MH348611)<br>Tulasnellaceae SSCDO-3 (MH348612)<br>Tulasnellaceae SSCDO-4 (MH348613)<br>Tulasnellaceae SSCDO-5 (MH348614)<br>Tulasnellaceae SSCDO-7 (MH348616)<br>Sebacinales SSCDO-6 (MH348615)	Guangxi	*D. officinale*	Protocorms	[65]
Tulasnella sp. JC02 (JN863900)<br>*Tulasnella* sp. JC05 (JN863903)	Yunnan	*D. nobile*	Roots	[66]
Sebacinaceae SHH44<br>Sebacinaceae SHH53	Guizhou	*D. officinale*	Germinated seeds	[67]
Epulorhiza sp. C_{20} (EF393629)<br>*Epulorhiza* sp. C_{36} (AJ313443)	Hunan	*D. officinale*	Root	[68]
Tulasnella sp. DOF18 (KX611641)	Zhejiang	*D. officinale*	Shoots	[69]
Mycena sp. F-23 (FJ544251)	Yunnan	*D. officinale*	Root	[70]
Tulasnella sp. S4 (KX587486)<br>*Tulasnella* sp. S5 (MN503661)<br>*Tulasnella* sp. S7 (JN863901)<br>*Sebacina* sp. S2 (HQ853682)<br>*Sebacina* sp. S3 (HQ853681)	Yunnan	*D. officinale*	Protocorms<br>or Root	[71]

References

19. Chen, D.Y.; Wang, X.J.; Li, T.Q.; Li, N.Q.; Gao, J.Y. In situ seedling baiting to isolate plant growth-promoting fungi from *Dendrobium officinale*, an over-collected medicinal orchid in China. *Glob. Ecol. Conserv*. **2021**, *28*, e01659.
24. Zi, X.M.; Sheng, C.L.; Goodale, U.M.; Shao, S.C.; Gao, J.Y. In situ seed baiting to isolate germination-enhancing fungi for an epiphytic orchid, *Dendrobium aphyllum* (Orchidaceae). *Mycorrhiza* **2014**, *24*, 487–499.
25. Huang, H.; Zi, X.M.; Lin, H.; Gao, J.Y. Host-specificity of symbiotic mycorrhizal fungi for enhancing seed germination, protocorm formation and seedling development of over-collected medicinal orchid, *Dendrobium devonianum*. *J. Microbiol*. **2018**, *56*, 42–48.
27. Meng, Y.Y.; Fan, X.L.; Zhou, L.R.; Shao, S.C.; Liu, Q.; Selosse, M.A.; Gao, J.Y. Symbiotic fungi undergo a taxonomic and functional bottleneck during orchid seeds germination: A case study on *Dendrobium moniliforme*. *Symbiosis* **2019**, *79*, 205–212.
28. Meng, Y.Y.; Shao, S.C.; Liu, S.J.; Gao, J.Y. Do the fungi associated with roots of adult plants support seed germination? A case study on *Dendrobium exile* (Orchidaceae). *Glob. Ecol. Conserv*. **2019**, *17*, e00582
29. Wang, X.J.; Wu, Y.H.; Ming, X.J.; Wang, G.; Gao, J.Y. Isolating ecological-specifc fungi and creating fungus-seed bags for epiphytic orchid conservation. *Glob. Ecol. Conserv*. **2021**, *28*, e01714.
30. Shao, S.C.; Burgess, K.S.; Cruse-Sanders, J.M.; Liu, Q.; Fan, X.L.; Huang, H.; Gao, J.Y. Using in situ symbiotic seed germination to restore over-collected medicinal orchids in Southwest China. *Front. Plant Sci*. **2017**, *8*, 888.
60. Chen, J.; Wang, H.; Guo, S.X. Isolation and identification of endophytic and mycorrhizal fungi from seeds and roots of *Dendrobium* (Orchidaceae). *Mycorrhiza* **2012**, *22*, 297–307. doi:10.1007/s00572-011-0404-0.
61. Gao, W.W.; Guo, S.X. Effect of three endophytic fungi on growth of *Dendrobium candidum* and *Anoectochilus roxburghii*. *Chin. Trad. Herb. Drug.* **2002**, *6*, 543–545. (In Chinese with English abstract).
62. Guo, S.X.; Cao, W.Q.; Gao, W.W. Isolation and biological activity of mycorrhizal fungi from *Dendrobium candidum* and *D. nobile*. *China J. Chin. Mater. Med.* **2000**, *25*, 338–341. (In Chinese with English abstract).
63. Shao, S.C.; Luo, Y.; Jacquemyn, H. Co-cultures of mycorrhizal fungi do not increase germination and seedling development in the epiphytic orchid *Dendrobium nobile*. *Front. Plant Sci.* **2020**, *11*, 571426. doi:10.3389/fpls.2020.571426.
64. Shao, S.C.; Wang, Q.X.; Beng, K.C.; Zhao, D.K.; Jacquemyn, H. Fungi isolated from host protocorms accelerate symbiotic seed germination in an endangered orchid species (*Dendrobium chrysotoxum*) from southern China. *Mycorrhiza* **2020**, *30*, 529–539. doi:10.1007/s00572-020-00964-w.
65. Shao, S.C.; Xi, H.P.; Mohandass, D. Symbiotic mycorrhizal fungi isolated via ex situ seed baiting induce seed germination of *Dendrobium catenatum* Lindl. (Orchidaceae). *Appl. Ecol. Environ. Res.* **2019**, *17*, 9753–9771. doi:10.15666/aeer/1704_97539771.
66. Tan, X.M.; Wang, C.L.; Chen, X.M.; Zhou, Y.Q.; Wang, Y.Q.; Luo, A.X.; Liu, Z.H.; Guo, S.X. In vitro seed germination and seedling growth of an endangered epiphytic orchid, *Dendrobium officinale*, endemic to China using mycorrhizal fungi (*Tulasnella* sp.). *Sci. Hortic.* **2014**, *165*, 62–68, doi:0.1016/j.scienta.2013.10.031.
67. Wang, H.; Fang, H.; Wang, Y.; Duan, L.; Guo, S.X. In situ seed baiting techniques in Dendrobium officinale Kimuraet Migo and Dendrobium nobile Lindl: The endangered Chinese endemic *Dendrobium* (Orchidaceae). *World J. Microbiol. Biotechnol.* **2011**, *27*, 2051–2059. doi:10.1007/s11274-011- 0667-9.
68. Wu, H.F.; Song, X.Q.; Hu, M.J. Screening and identification of endophytic and probiotic fungi of *Dendrobium catenatum*. *J. SW. Forest. Univer.* **2011**, *31*, 47–52. (In Chinese with English abstract).

69. Wu, L.S.; Dong, W.G.; Si, J.P.; Liu, J.J.; Zhu, Y.Q. Endophytic fungi, host genotype, and their interaction influence the growth and production of key chemical components of *Dendrobium catenatum*. *Fungal Biol.* **2020**, *124*, 864–876. doi:10.1016/j.funbio.2020.07.002.
70. Zhang, L.C.; Chen, J.; Lv, Y.L.; Gao, C.; Guo, S.X. *Mycena* sp., a mycorrhizal fungus of the orchid *Dendrobium officinale*. *Mycol. Progress* **2012**, *11*, 395–401. doi:10.1007/s11557-011-0754-1.
71. Zhang, Y.; Li, Y.Y.; Chen, X.M.; Guo, S.X.; Lee, Y.I. Effect of different mycobionts on symbiotic germination and seedling growth of Dendrobium officinale, an important medicinal orchid. *Bot Stud.* **2020**, *61*, 2. doi:10.1186/s40529-019-0278-6.
